# Supplementary material for: Assessment of antidiabetic, hepatoprotective, and analgesic effects of quinazolinone derivative, (E)-1-Benzoyl-3-((4- (Dimethylamino) Benzylidene) Amino)-2-(4-(Dimethylamino) Phenyl)-2,3 dihydroquinazoline-4(1h)-one, in diabetes induced mice model
Source: PLoS One. 2026 Feb 4;21(2):e0327627. doi: 10.1371/journal.pone.0327627 (PMC12977029; doi:10.1371/journal.pone.0327627)
Supplement: Attachment — Submitted filename: Response to reviwers comments.docx [file pone.0327627.s001.docx]

# Response to Editor and Reviewer Comments

## Section 1: Response to Editor’s General and Journal Requirements

1. Manuscript style and format requirements

We have carefully revised the manuscript according to the PLOS ONE formatting style. The manuscript now follows the journal’s structure, file naming conventions, and style template guidelines. All figures, tables, and references have been reformatted to meet journal standards.

2. Ethics statement placement

The ethics statement has been retained only within the Methods section, and removed from any other sections as instructed.

3. Data Availability Statement

We have added a complete Data Availability Statement as per PLOS ONE policy: “All data are available within the manuscript and/or supporting information files.”

4. Data sharing plan

We confirm that all underlying data supporting our findings are now freely accessible as Supporting Information files. No ethical or participant privacy concerns prevent data sharing.

5. Competing Interests

We have completed the Competing Interests section with the statement: “The authors have declared that no competing interests exist.”

6. Title consistency

The title in the manuscript and on the submission system have been made identical.

7. Data anonymization

All personal or identifiable data have been removed from the dataset. The uploaded dataset is fully anonymized and complies with PLOS data policy.

8. Supporting Information captions

Captions for all Supporting Information files have been included at the end of the manuscript, and corresponding in-text citations have been updated accordingly.

9. Suggested citations

We have reviewed the literature recommended by reviewers and cited relevant works where appropriate.

10. Reference list accuracy

The reference list has been thoroughly reviewed and corrected for formatting, accuracy, and completeness. No retracted articles are included.

## Section 2: Response to Reviewer #1 Comments

1. Chemicals and Equipment section

We thank the reviewer for these valuable suggestions. The section title has been corrected to “Chemicals and Equipment.” We have categorized the list clearly under reagents and instruments. The test compound is now fully described with its chemical name, purity (≥98%), and preparation details.

2. Animals section

We standardized all spelling to British English for consistency and removed unnecessary details regarding test timing. The subheading has been revised to 'Animal Care and Use,' and ethical compliance is clearly described.

3. Acute Toxicity section

Behavioral observations are now described using standardized terminology (e.g., 'tremors', 'locomotor activity', 'grooming'). Quantitative mortality data have been included. Grammatical corrections were made.

4. Diabetes Induction and Glucose Monitoring

Baseline glucose levels have been added to the Results section. The glucometer brand/model (Accu-Chek Active, Roche Diagnostics) is now specified. The criteria for diabetes induction (≥250 mg/dL) are clarified, and validation details have been included.

5. Preclinical Pharmacological Tests

The percentage analgesia formula has been reformatted, and an appropriate citation is added. The term 'peripheral agony' has been replaced with 'nociceptive stimulus.' We clarified animal numbers per group (n=6), randomization procedures, and blinding during analysis. The section has been restructured with clearer subheadings.

6. Histological and Biochemical Analysis

The centrifugation speed has been corrected to 3000 rpm. High-resolution histological images have been added as Supporting Information. We have described ANOVA assumptions and validation steps explicitly.

7. Statistical Analysis

We now specify the post-hoc test used (Tukey’s multiple comparison). Details of software versions (SPSS v25, GraphPad Prism v9.0) and data normality checks have been added.

8. General Suggestions

We implemented all general improvements recommended by the reviewer, including: (i) better subheadings, (ii) consistent scientific tone, (iii) clear reporting of sample sizes and euthanasia, and (iv) inclusion of an experimental timeline flowchart in Supporting Information.

## Section 3: Summary of Revisions

1. Manuscript revised to comply fully with PLOS ONE format and ethical/data policies.
2. Improved clarity and organization across all Methods sections.
3. Added missing experimental details, including animal group sizes, statistical validation, and glucometer specifications.
4. Updated histological images and added Supporting Information captions.
5. Corrected all linguistic inconsistencies and improved readability.
6. Revised Data Availability, Ethics, and Competing Interests statements as per journal policy.
