# Supplementary material for: Assessment of antidiabetic, hepatoprotective, and analgesic effects of quinazolinone derivative, (E)-1-Benzoyl-3-((4- (Dimethylamino) Benzylidene) Amino)-2-(4-(Dimethylamino) Phenyl)-2,3 dihydroquinazoline-4(1h)-one, in diabetes induced mice model
Source: PLoS One. 2026 Feb 4;21(2):e0327627. doi: 10.1371/journal.pone.0327627 (PMC12977029; doi:10.1371/journal.pone.0327627)
Supplement: Attachment — Submitted filename: Point by Point response.docx [file pone.0327627.s002.docx]

**RESPONSE TO REVIEWER COMMENTS**

**Manuscript.ID:**PONE-D-25-32082R1
**Title:** *Assessment of Antidiabetic, Hepatoprotective, and Analgesic Effects of Quinazolinone Derivative, (E)-1-Benzoyl-3-((4-(Dimethylamino) Benzylidene) Amino)-2-(4-(Dimethylamino) Phenyl)-2,3-dihydroquinazoline-4(1H)-one, in Diabetes-Induced Mice Model*

We thank the Academic Editor and Reviewer #1 for their valuable time and constructive feedback. All comments have been carefully addressed, and corresponding revisions are highlighted in the “Revised Manuscript with Track Changes.” Below, each reviewer comment is reproduced in italics, followed by our detailed response.

**Reviewer #1**

**1. Structural and Formatting Issues**

**Comment:**
*Inconsistent section headings; redundant subsections “Animals” and “Animals care.”*

**Response:**
We have standardized all section headings according to PLOS ONE formatting.

- “Chemicals and Equipments” → changed to “Chemicals and Equipment.”
- The subsections “Animals” and “Animals care” have been merged into a single section titled “Animal Care and Ethical Approval.”
- Uniform capitalization and formatting have been applied throughout *Materials and Methods.*

**2. Language and Grammar**

**Comment:**
*Multiple grammatical and phrasing issues (e.g., “Chemicals and Equipments,” “Histological study were done,” “The mice’s were,” “Peripheral agony,” “Rumpled with a safety pin”).*

**Response:**
The entire manuscript has been thoroughly revised for grammar, punctuation, and scientific phrasing. Examples of key corrections:

- “Chemicals and Equipments” → “Chemicals and Equipment.”
- “Histological study were done” → “Histological studies were conducted”
- “The mice’s were then observed” → “The mice were then observed”
- “Peripheral agony” → “Peripheral pain (nociceptive response).”
- “Rumpled with a safety pin” → “Stimulated using a calibrated safety pin.”
  We also used *Grammarly* and manual proofreading by two co-authors to ensure clarity and professional tone.

**3. Scientific Rigor and Clarity**

**Comment**
*Chemical concentrations and preparation methods missing.*

**Response:**
All chemical preparation details have been added in *Materials and Methods → Chemicals and Reagents.*
For instance, alloxan was freshly dissolved in sterile normal saline (0.9 % NaCl) at a concentration of 150 mg/kg before injection. Similar clarifications were added for other reagents.

**Comment** *Environmental conditions for animal housing not described.*

**Response:**
We have now included: “ The animals were kept in well-ventilated housing maintaining temperature at 26 ^0^ C and humidity between 30% and 70% and provided with 12:12 hour light-dark cycle to support the circadian rhythms of the animals. The housing was also supplied with dry wood shavings used as bedding, which were regularly changed to maintain a hygienic and microbe-free environment.”

**Comment :***Sample sizes missing throughout.*

**Response:**
Sample sizes for each experimental group (n = 5 mice/group) are now clearly stated in the text and figure legends. Statistical power was verified to ensure adequate sensitivity.

**Comment**
*Ambiguity in toxicity and analgesia descriptions.*

**Response:**
The *Acute Toxicity* section has been expanded: “Safety was assessed by absence of mortality, changes in behavior, grooming, feeding, or mobility for 14 days post-administration.”
In *Analgesia Methods,* the formula now defines “cut-off time” as “the maximum time (15 s) beyond which the test was terminated to avoid tissue injury.”

**4. Ethical and Regulatory Compliance**

**Comment:**
*Ethical statement should confirm adherence to international standards; euthanasia description too graphic.*

**Response:** Details included as: Ethical committee of the Pharmacy Department of the University of Peshawar approved experimental methods (approval number 06/EC-FLES-UOP/2023). The testing period was from 8:00 am to 5:00 pm. The Scientific Procedure Act of U.K. 1986 and The ARRIVE Guidelines 2.0 were followed when doing procedures on animals.”

**5. Statistical Analysis**

**Comment:**
*Statistical details (p-values, post-hoc tests) are insufficient.*

**Response:**

The *Statistical Analysis* section now includes: “Data are expressed as mean ± SEM. Statistical significance was determined using one-way ANOVA followed by Tukey’s post-hoc test. Significance threshold was set at p < 0.05. All analyses were performed using GraphPad Prism v10.0. Confidence intervals (95 %) were calculated where applicable.”

**6. Referencing and Citation**

**Comment:**
*Inconsistent citation format; missing bibliography; methods not attributed to original sources.*

**Response:**
A complete reference list has been compiled in accordance with PLOS ONE style.
All methodological procedures (e.g., Von Frey, hot-plate, and acetic-acid writhing tests) now include appropriate citations to the original or validated sources.
In-text citations are numbered sequentially, and all references are listed at the end of the manuscript.

**7. Recommendations for Improvement**

**Comment:**
*Revise for clarity and grammar, merge redundant subsections, add details, expand ethics and statistics, ensure proper citation.*

**Response:**
All recommendations have been fully implemented:

- Grammar and clarity improved across all sections.
- Redundant subsections merged.
- Detailed methodological parameters (sample sizes, preparation steps, environmental conditions) added.
- Ethical and statistical statements expanded.
- References standardized and verified.

We sincerely thank the reviewer for these comprehensive suggestions that have substantially enhanced the manuscript’s quality and compliance with PLOS ONE standards.

We trust that the revised version addresses all reviewer concerns. We are grateful for the editor’s opportunity to revise and resubmit, and we hope that our responses and improvements will now make the manuscript suitable for publication in *PLOS ONE.*
